# Supplementary material for: Validation of a New Duplex Real-Time Polymerase Chain Reaction for Chlamydia trachomatis DNA Detection in Ocular Swab Samples
Source: Diagnostics (Basel). 2024 Apr 25;14(9):892. doi: 10.3390/diagnostics14090892 (PMC11083659; doi:10.3390/diagnostics14090892)
Supplement: Supplementary file 1 [file diagnostics-14-00892-s001.zip › Favacho_Supplementary Figures (FS1-FS5).pptx]

## Slide 1
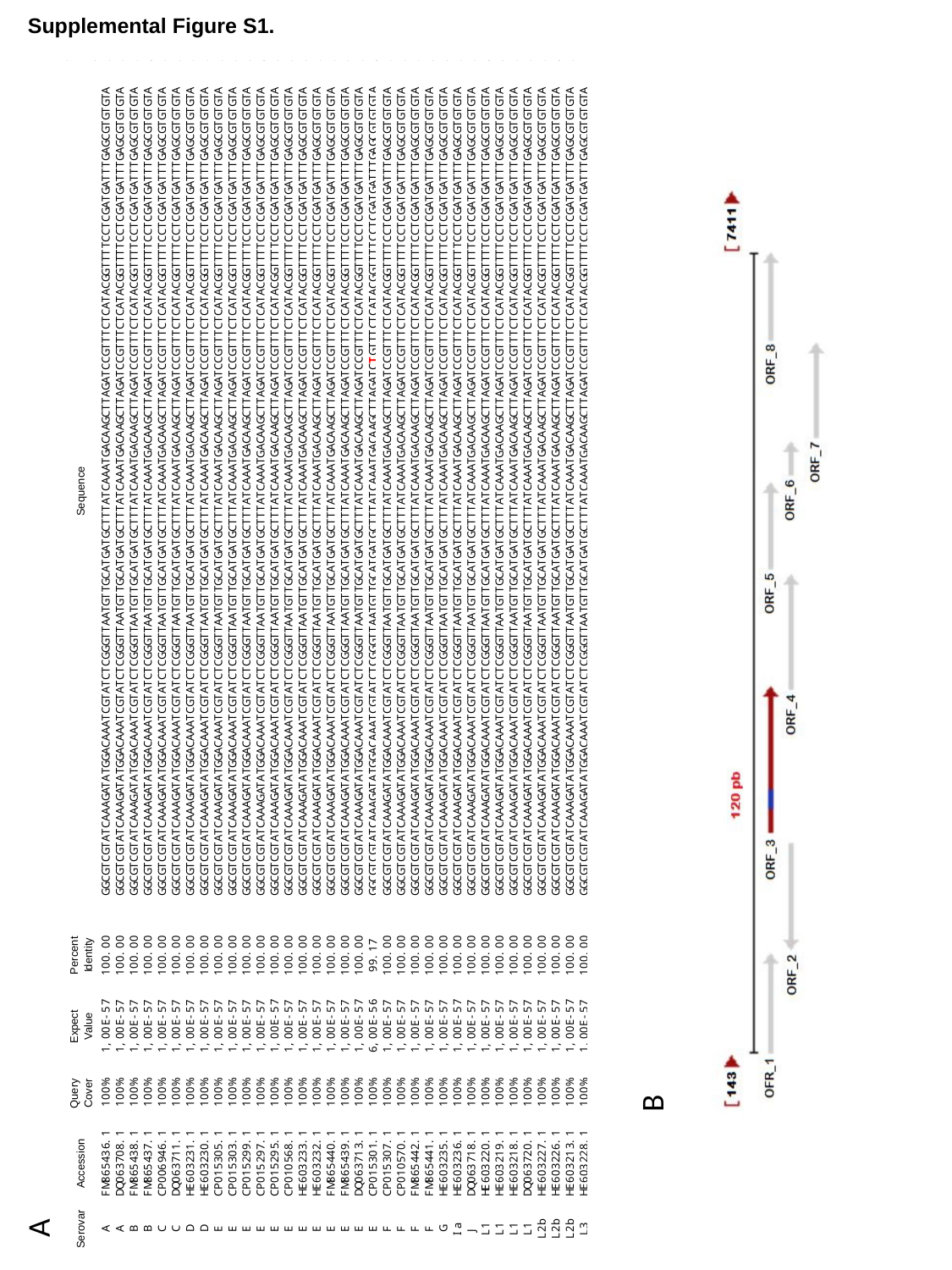

Supplemental Figure S1.
B
A

## Slide 2
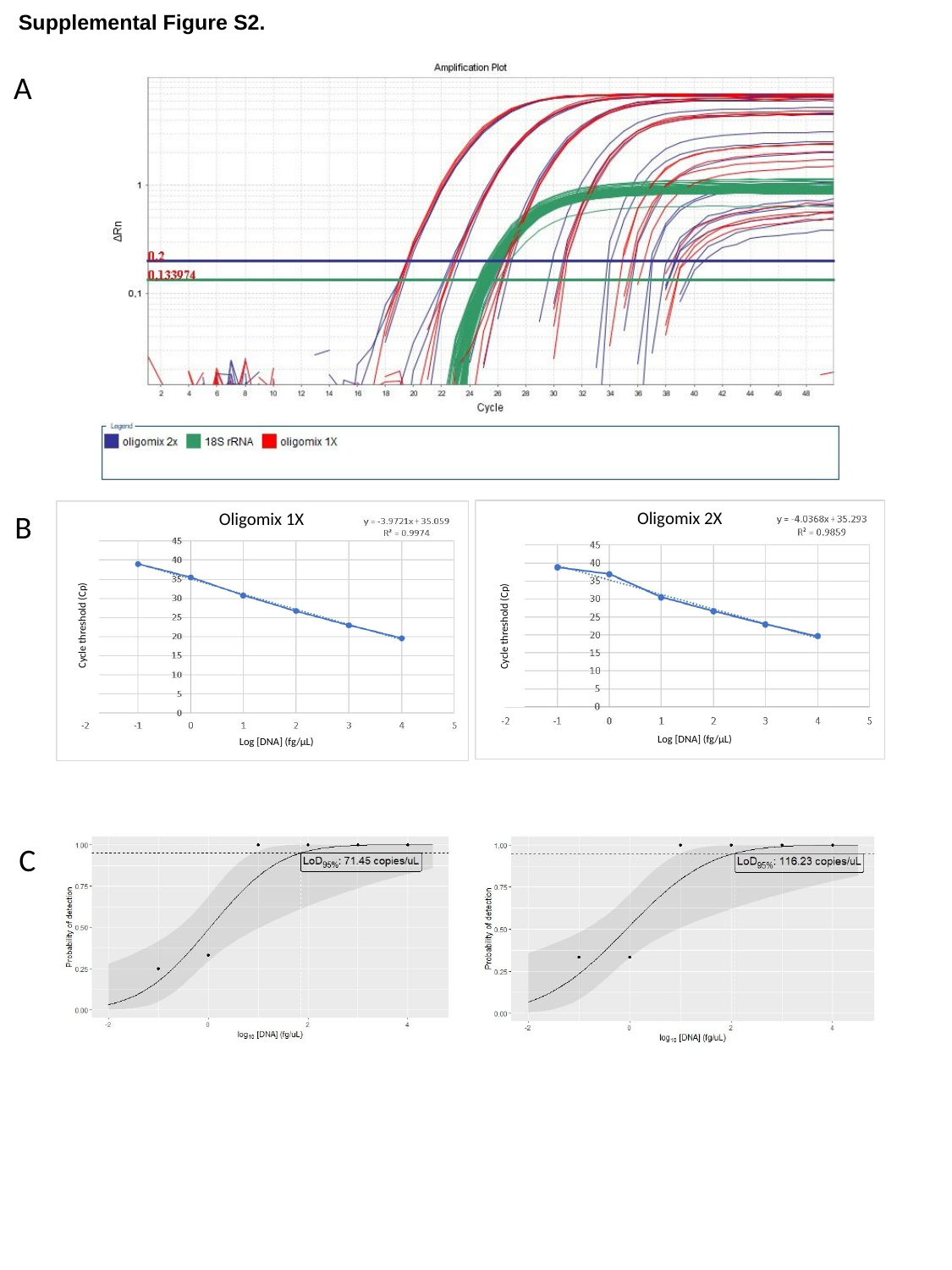

Supplemental Figure S2.
A
Oligomix 2X
B
Oligomix 1X
Cycle threshold (Cp)
Cycle threshold (Cp)
Log [DNA] (fg/µL)
Log [DNA] (fg/µL)
C

## Slide 3
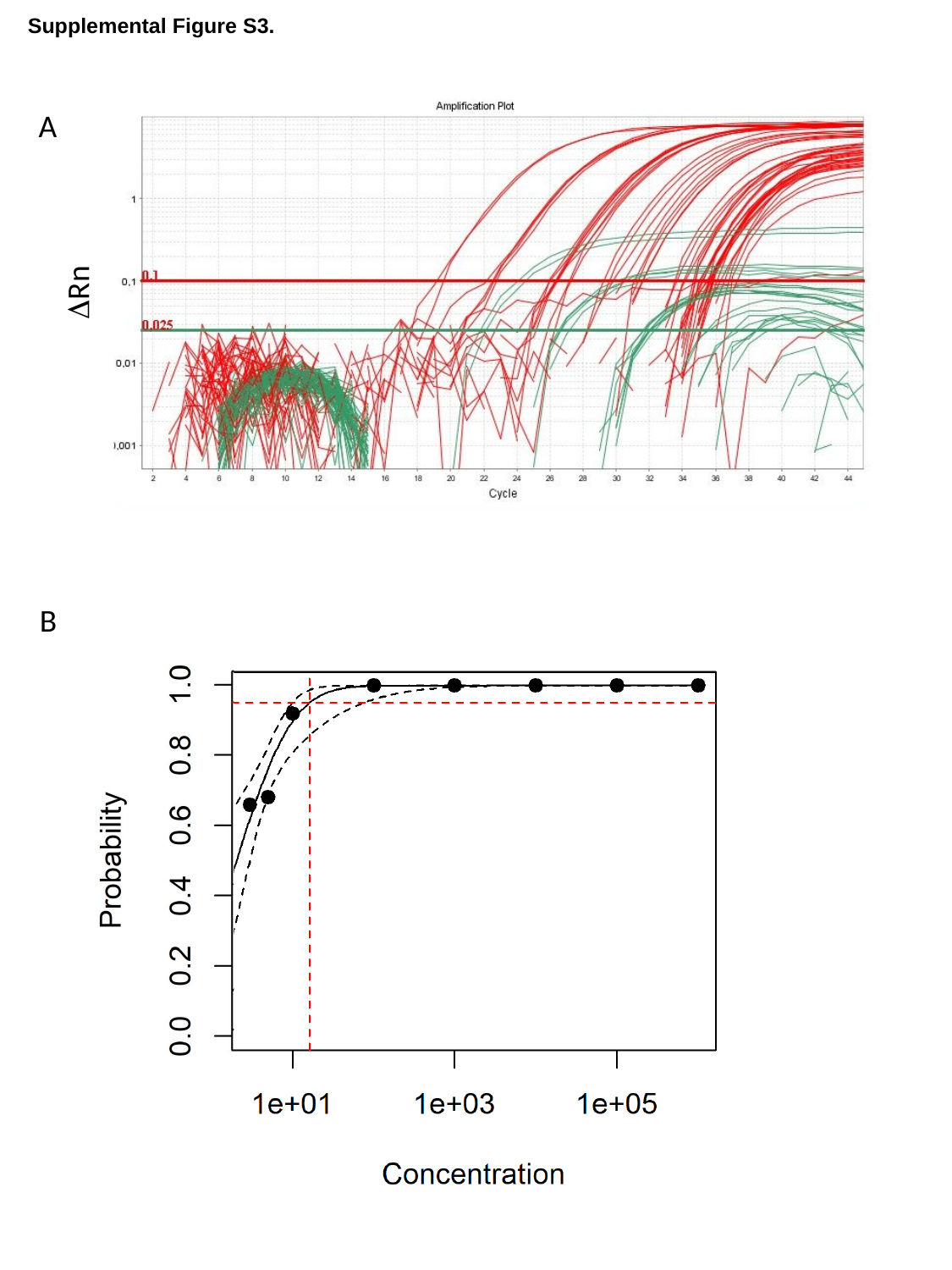

Supplemental Figure S3.
A
Panel A
DRn
B

## Slide 4
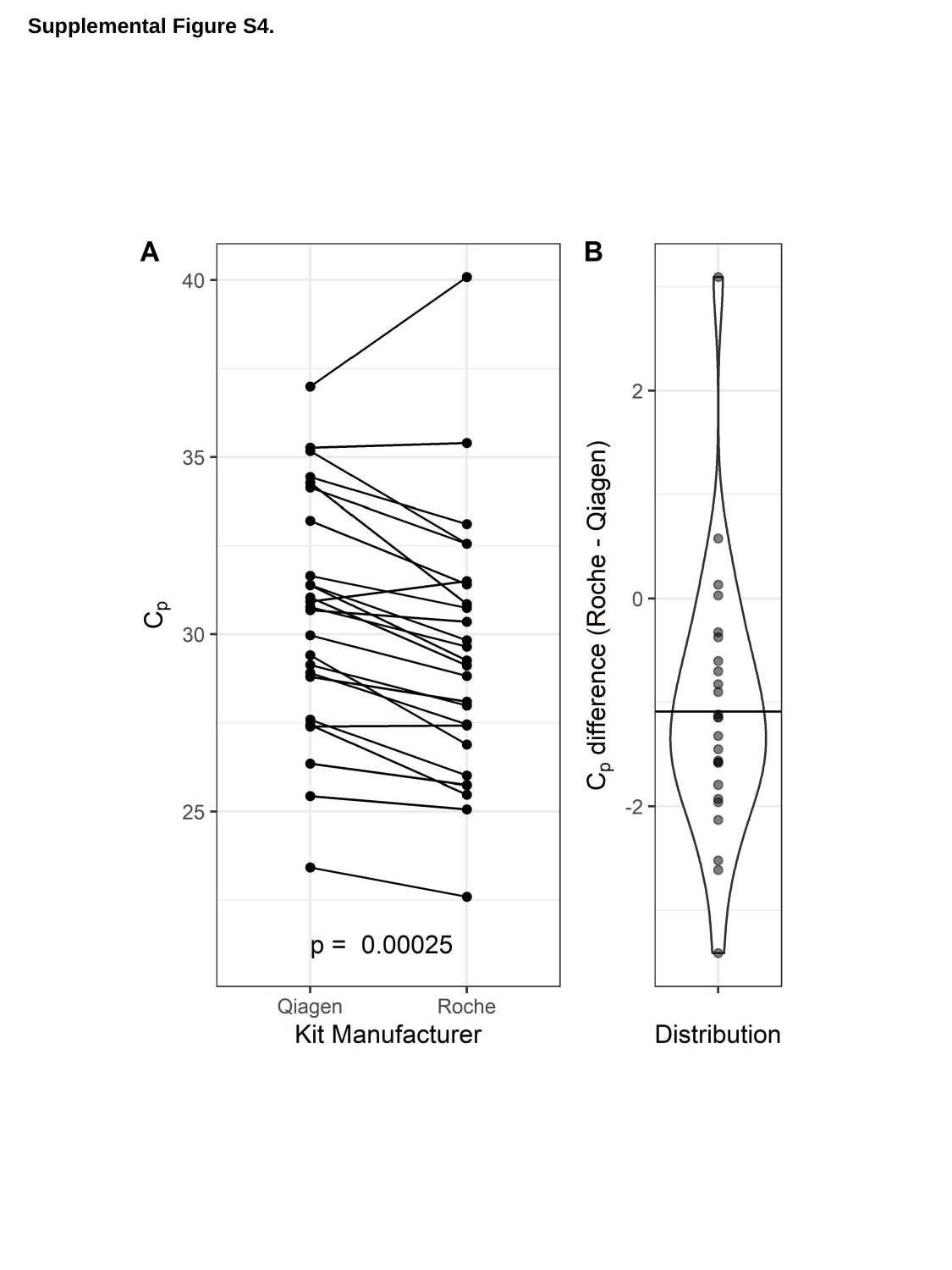

Supplemental Figure S4.

## Slide 5
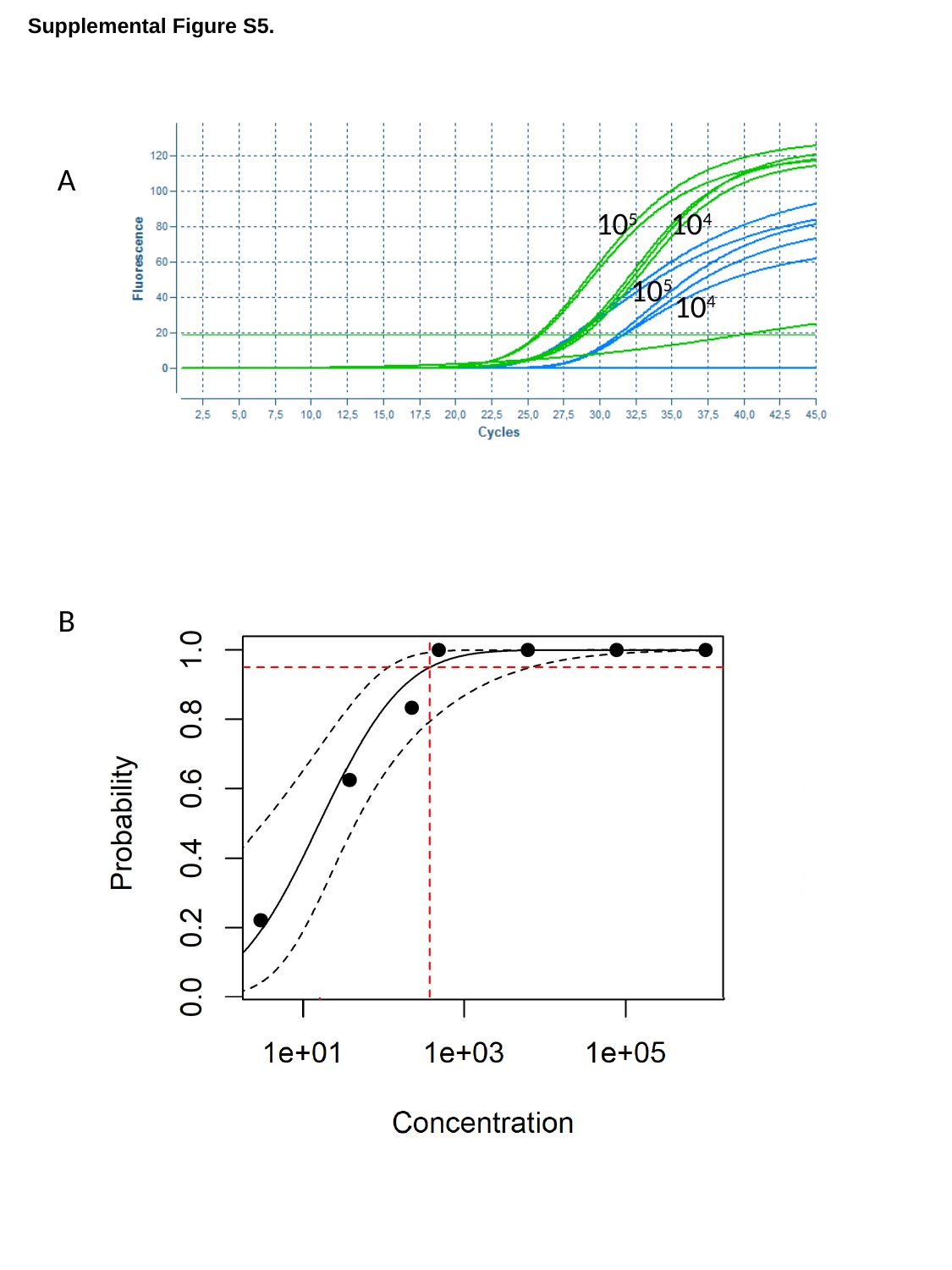

Supplemental Figure S5.
A
105
104
105
104
B
