# Supplementary material for: Validation of a New Duplex Real-Time Polymerase Chain Reaction for Chlamydia trachomatis DNA Detection in Ocular Swab Samples
Source: Diagnostics (Basel). 2024 Apr 25;14(9):892. doi: 10.3390/diagnostics14090892 (PMC11083659; doi:10.3390/diagnostics14090892)
Supplement: Supplementary file 1 [file diagnostics-14-00892-s001.zip › Supplemental Tables.pdf]

Supplemental Table S1. List of 55 organisms tested for unspecific amplification against all six oligonucleotides described in Methods. NA = not available.

Species	Strain
Plasmodium falciparum	W2
Trypanosoma cruzi	Dm28c
Leishmania guyanensis	NA
Leishmania amazonensis	NA
Leishmania braziliensis	NA
Leishmania infantum	NA
Bordetella pertussis	Tohama I (ATCC BAA589D)
Bordetella parapertussis	12822 (ATCC BAA587D)
Staphyloccocus aureus	MRSA C1A2
Staphyloccocus aureus	MRSA C1A3
Enterococcus faecalis	EFC C1A4
Enterococcus faecalis	EFC C1A6
Enterococcus faecium	EFA VANA
Enterococcus faecium	EFA VANB
Staphylococcus haemolyticus	SHA 31
Staphylococcus haemolyticus	SHA 35
Acinetobacter baumannii	ABA 28
Acinetobacter baumannii	ABA 39
Streptococcus agalactidae	EGB C1A6
Streptococcus agalactidae	EGB C1A10
Staphylococcus epidermidis	SEP 8
Staphylococcus epidermidis	SEP 10
Stenotrophomonas maltophilia	SML 440
Stenotrophomonas maltophilia	SML 6430
Enterobacter cloacae	ECL 254
Enterobacter cloacae	ECL 407
Proteus mirabilis	PMI 95
Proteus mirabilis	PMI 341
Serratia marcescens	SMA 27

Serratia marcescens	SMA 290
Enterobacter aerogenes	EA 16
Enterobacter aerogenes	EA 35
Klebsiella oxytoca	KOX 204
Klebsiella oxytoca	KOX 635
Morganella morganii	MMO 46C
Streptococcus pyogenes	EGA ERMA
Streptococcus pyogenes	EGA ERMB
Streptococcus pyogenes	LMO 384L
Streptococcus pyogenes	LMO 394L
Listeria monocytogenes	SPY R4
Listeria monocytogenes	SPY R5
Citrobacter freundii	CFR 866
Pseudomonas aeruginosa	PAE 8
Candida albicans	CA 296
Candida albicans	CA297
Candida paropsilosis	CPA 140
Candida paropsilosis	CPA 159
Candida glabrata	CGL 56
Candida glabrata	CGL 57
Candida tropicalis	CTR 76
Candida tropicalis	CTR 81
Candida krusei	CK 27
Candida krusei	CK 28
Aspergilus fumigatus	AFU 71
Aspergilus fumigatus	AFU 75

Supplemental Table S2. MIQE checklist.

ITEM TO CHECK	IMPORTANCE	CHECKLIST
**EXPERIMENTAL DESIGN**		
Definition of experimental and control groups	**E**	1. Experimental groups: 62 samples (50 from endemic area and 12 from non-endemic area) 2. Control groups: Extracted commercial DNA; Synthetic double strand DNA; 50 pre-characterized samples (30 positive and 20 negative)
Number within each group	**E**	Yes (see above)
Assay carried out by core lab or investigator's lab?	D	Investigator's lab
Acknowledgement of authors' contributions	D	Yes
**SAMPLE**		
Description	**E**	Eyelid samples collected using dry swabs
Volume/mass of sample processed	D	Two hundred microliters
Microdissection or macrodissection	**E**	Not performed.
Processing procedure	**E**	Swabs were placed in preserving gel, according to manufacturer's instructions
If frozen - how and how quickly?	**E**	Samples were not frozen.
If fixed - with what, how quickly?	**E**	Samples were not fixed.
Sample storage conditions and duration (especially for FFPE samples)	**E**	Samples were stored in refrigerator until DNA extraction. Extracted DNA was kept in -20 ^{o}C freezer.
**NUCLEIC ACID EXTRACTION**		
Procedure and/or instrumentation	**E**	Silica-based spin collumns
Name of kit and details of any modifications	**E**	QIAmp DNA Blood Mini kit (Qiagen, Germany) and High Pure PCR Template Preparation kit (Roche Applied Sciences, Germany)

Source of additional reagents used	D	Yes
Details of DNase or RNAse treatment	**E**	Not performed.
Contamination assessment (DNA or RNA)	**E**	Contamination by proteins was assessed spectrophotometrically.
Nucleic acid quantification	**E**	Yes
Instrument and method	**E**	Nanodrop 2000c; spectrometry
Purity (A260/A280)	D	Not reported.
Yield	D	Not reported.
RNA integrity method/instrument	**E**	Not applicable.
RIN/RQI or Cq of 3' and 5' transcripts	**E**	Not applicable.
Electrophoresis traces	D	Not applicable.
Inhibition testing (Cq dilutions, spike or other)	**E**	Cq values for the human control target out of specified ranges were interpreted as inhibition of the reaction or failure of DNA extraction, and samples DNA were re-extracted. Non-template controls (NTC, molecular grade water) as well as trachoma-negative human DNA were routinely used as controls.
**REVERSE TRANSCRIPTION**		
Complete reaction conditions	**E**	Not applicable.
Amount of RNA and reaction volume	**E**	Not applicable.
Priming oligonucleotide (if using GSP) and concentration	**E**	Not applicable.
Reverse transcriptase and concentration	**E**	Not applicable.
Temperature and time	**E**	Not applicable.
Manufacturer of reagents and catalogue numbers	D	Not applicable.
Cqs with and without RT	D*	Not applicable.
Storage conditions of cDNA	D	Not applicable.
**qPCR TARGET INFORMATION**		

If multiplex, efficiency and LOD of each assay.	**E**	*C. trachomatis* DNA detection (ABI7500): efficiency of 92% (slope –3.52) and LOD95% of 13.04 genome equivalents per reaction
Sequence accession number	**E**	*C. trachomatis* cryptic plasmid DNA (accession number) Human 18S rRNA gene (accession number)
Location of amplicon	D	Yes, Supplementary Figure S1
Amplicon length	**E**	120 bp
In silico specificity screen (BLAST, etc)	**E**	Yes
Pseudogenes, retropseudogenes or other homologs?	D	--
Sequence alignment	D	Supplementary Figure S1
Secondary structure analysis of amplicon	D	--
Location of each primer by exon or intron (if applicable)	**E**	Not performed.
What splice variants are targeted?	**E**	Not performed.
**qPCR OLIGONUCLEOTIDES**		
Primer sequences	**E**	cryp05F 5'-GGCGTCGTATCAAAGATATGG-3'<br>cryp06R 5'-CGATGATTTGAGCGTGTGTA-3'<br>18S2F 5'-GAAACTGCGAATGGCTCATTAAATCA-3'<br>18S2R 5'-AGAGCTAATACATGCCGACGGG-3'
RTPrimerDB Identification Number	D	--
Probe sequences	D**	cryp11P 5'-FAM-TCTCGGGTTAATGTTGCATGATGCTT-BHQ1 - 3'<br>18S2P 5'-HEX- TGGTTCCTTTGGTCGCTCGCTCC-BHQ1-3'
Location and identity of any modifications	**E**	Not applicable.
Manufacturer of oligonucleotides	D	IDT DNA Technologies (Coralville, USA)
Purification method	D	reverse phase and HPLC
**qPCR PROTOCOL**		

Complete reaction conditions	**E**	Detection of *C. trachomatis* cryptic plasmid and human 18S rRNA gene DNA was performed using the Multiplex PCR Mastermix (IBMP, Curitiba, Brazil) containing Taq DNA polymerase, 9 mM Mg-acetate, 0.8 mM dNTP (Thermo Scientific, USA), 5 µL of extracted DNA, oligonucleotides cryp05F (0.2 µM), cryp06R (0.2 µM), cryp11P (0.1 µM), 18S2F (0.1 µM), 18S2R (0.1 µM), and 18S2P (0.05 µM), in a reaction volume of 25 µL.
Reaction volume and amount of cDNA/DNA	**E**	Reaction volume of 25 µL and 5 µL of extracted DNA
Primer, (probe), Mg++ and dNTP concentrations	**E**	cryp05F and cryp06R: 0.2 µM; cryp11P: 0.1 µM 18S2F and 18S2R: 0.1 µM; 18S2P: 0.05 µM 9 mM Mg-acetate 0.8 mM dNTP
Polymerase identity and concentration	**E**	Taq DNA polymerase
Buffer/kit identity and manufacturer	**E**	Inst Biologia Molecular do Parana (IBMP, Curitiba, Brazil)
Exact chemical constitution of the buffer	D	--
Additives (SYBR Green I, DMSO, etc.)	**E**	Yes. None used.
Manufacturer of plates/tubes and catalog number	D	--
Complete thermocycling parameters	**E**	95˚C/10 min, and 45 x [95˚C/15 sec + 60˚C/1 min]], using ROX as passive fluorescence. Baseline was set from 3 to 15 for both targets. For C. trachomatis DNA, threshold was set to 0.1, and quantification cycle (Cq) values between 19 and 41.50 were considered positive detections. For human DNA, threshold was set to 0.1 and detections were considered positive and free of contaminants if Cq values were between 14 and 37.
Reaction setup (manual/robotic)	D	Manual
Manufacturer of qPCR instrument	**E**	Thermo Fisher Scientific – Waltham, USA

**qPCR VALIDATION**		
Evidence of optimisation (from gradients)	D	--
Specificity (gel, sequence, melt, or digest)	**E**	qPCR was validated using samples previously categorized by a distinct qPCR assay (target and instrument). Results are shown in Table II.
For SYBR Green I, Cq of the NTC	**E**	Not applicable.
Standard curves with slope and y-intercept	**E**	C. trachomatis DNA detection (ABI7500): slope –3.52, Y-intercept of -32.79
PCR efficiency calculated from slope	**E**	Efficiency of 92%
Confidence interval for PCR efficiency or standard error	D	Not shown.
r2 of standard curve	**E**	98.6% (ABI7500)
Linear dynamic range	**E**	10^-18 to 10^-12 g/µL DNA
Cq variation at lower limit	**E**	SD of 0.91 Ct at lower limit (10 ag/µL)
Confidence intervals throughout range	D	Not shown.
Evidence for limit of detection	**E**	Probit calculations, described in Methods and Results.
If multiplex, efficiency and LOD of each assay.	**E**	*C. trachomatis* DNA detection (ABI7500): efficiency of 92% (slope –3.52) and LOD95% of 13.04 genome equivalents per reaction
**DATA ANALYSIS**		
qPCR analysis program (source, version)	**E**	ABI7500 software v2.0.6
Cq method determination	**E**	Baseline was set from 3 to 15 for both targets. For *C. trachomatis* DNA, threshold was set to 0.1. For human DNA, threshold was set to 0.1.
Outlier identification and disposition	**E**	For *C. trachomatis* DNA, quantification cycle (Cq) values between 19 and 41.50 were considered positive detections. For human DNA, detections were considered positive and free of contaminants if Cq values were between 14 and 37.
Results of NTCs	**E**	Non-template controls should show no amplification for both targets, whereas trachoma-negative controls

		should show positive detections for human DNA with Cq values were between 14 and 37.
Justification of number and choice of reference genes	**E**	18S rRNA is a common reference gene for human samples.
Description of normalisation method	**E**	ROX as passive fluorescence
Number and concordance of biological replicates	D	--
Number and stage (RT or qPCR) of technical replicates	**E**	All qPCR assays in the ABI7500 system were performed in technical triplicates, except for LOD determination (8-12 replicates).
Repeatability (intra-assay variation)	E	Repeatability was found to be <8.8% (highest at 0.1 fg/µL) when assayed by three independent experienced operators.
Reproducibility (inter-assay variation, %CV)	D	Reproducibility (inter-assay variation) was found to be <5.8% (highest at 0.03 fg/µL) when assayed by three independent experienced operators.
Power analysis	D	Not performed.
Statistical methods for result significance	**E**	Bland-Altman analyses and Kappa coefficient calculations
Software (source, version)	E	R scripts
Cq or raw data submission using RDML	**D**	--

Supplemental Table S3. Data used for calculating the LOD95% using commercial DNA for *C. trachomatis* serovar J in the ABI7500 instrument. Concentrations are shown as fg/μL. NEG = negative

10^4	10^3	10^2	10^1	10^0	10^-1			3x10^-2		
18.57	21.87	26.05	30.14	35.00	37.29	NEG	NEG	NEG	NEG	NEG
18.81	22.03	25.76	30.16	33.64	37.28	35.88	NEG	NEG	34.70	NEG
18.83	22.42	26.20	30.10	33.93	35.67	35.82	NEG	NEG	37.18	NEG
18.44	21.23	25.77	30.20	34.29	NEG	NEG	35.96	36.35	NEG	NEG
18.27	21.32	25.67	29.92	34.09	35.72	37.18	35.17	NEG	NEG	NEG
18.46	21.77	25.57	30.30	34.06	36.59	36.99	38.23	NEG	NEG	NEG
18.12	21.44	24.75	30.10	34.52	37.36	37.22	35.66	NEG	NEG	NEG
18.44	21.71	25.02	29.68	33.47	33.67	NEG	NEG	NEG	NEG	NEG
18.61	21.59	25.50	29.93	35.42	39.62	NEG	NEG	NEG	NEG	NEG
18.56	22.07	25.68	30.09	32.82	39.01	NEG	NEG	NEG	NEG	NEG
18.32	21.43	25.77	29.78	33.24	36.71	NEG	NEG	38.13	NEG	NEG
18.71	24.65	16.96	29.87	35.32	36.29	NEG	NEG	NEG	NEG	41.64
18.33	14.57	25.19	30.49	34.03	40.55	NEG	NEG	NEG	NEG	NEG
18.56	21.94	25.73	30.44	35.02	36.01	35.82	NEG	NEG	NEG	NEG
18.42	21.68	25.75	30.22	34.14	37.60	38.95	NEG	NEG	39.23	NEG
18.67	22.15	25.77	30.18	34.88	NEG	NEG	NEG	NEG	NEG	NEG
18.62	21.91	25.94	29.28	35.18	NEG	NEG	38.86	NEG	NEG	NEG
18.64	22.22	25.58	30.40	33.65	NEG	NEG	NEG	42.64	NEG	40.07
18.75	21.80	26.24	29.88	33.22	NEG	NEG	36.32	NEG	NEG	NEG

Supplemental Table S4. Data used for calculating the LOD95% using synthetic DNA (gBlock®) in the ABI7500 instrument. Concentrations are shown as copies/µL. NEG = negative.

10^6	10^5	10^4	10^3	10^2	10^1	5x10^0	3x10^0
16.02	19.41	22.20	27.67	31.02	33.25	35.40	35.15
16.20	19.92	23.11	27.62	31.46	32.61	37.14	35.72
NEG	19.49	23.05	27.77	32.18	36.26	34.62	35.12
NEG	19.33	23.26	27.52	31.56	35.69	35.88	35.72
NEG	NEG	22.15	27.42	30.37	36.00	35.66	36.89
NEG	NEG	22.75	27.12	29.08	NEG	36.11	35.15
NEG	NEG	22.55	28.09	31.89	33.64	33.62	35.64
NEG	NEG	22.57	27.58	30.99	36.39	37.15	34.69
NEG	NEG	NEG	26.04	31.71	34.59	35.59	NEG
NEG	NEG	NEG	26.40	31.51	37.29	35.46	36.17
NEG	NEG	NEG	26.39	29.89	33.46	35.83	35.95
NEG	NEG	NEG	26.13	29.21	35.41	34.19	34.86
NEG	NEG	NEG	27.54	30.62	35.21	36.85	38.30
NEG	NEG	NEG	27.00	31.19	34.09	36.85	35.59
NEG	NEG	NEG	26.66	30.60	34.80	NEG	35.51
NEG	NEG	NEG	26.76	30.58	38.73	NEG	36.69
NEG	NEG	NEG	26.85	30.63	34.42	37.62	NEG
NEG	NEG	NEG	27.43	31.19	34.52	NEG	34.91
NEG	NEG	NEG	27.76	31.40	34.86	35.72	NEG
NEG	NEG	NEG	25.70	31.53	35.38	35.43	NEG
NEG	NEG	NEG	27.36	31.01	NEG	37.32	35.11
NEG	NEG	NEG	27.36	29.43	.	35.32	36.47

NEG	NEG	NEG	27.85	32.01	35.03	.	36.56
NEG	NEG	NEG	27.71	31.22	34.51	.	36.42
NEG	NEG	NEG	26.93	30.58	34.63	35.34	.
NEG	NEG	NEG	27.09	30.91	34.85	NEG	.
NEG	NEG	NEG	27.01	30.52	34.93	NEG	.
NEG	NEG	NEG	27.53	30.90	36.67	37.19	.
NEG	NEG	NEG	27.01	31.44	35.60	NEG	.
NEG	NEG	NEG	26.93	30.51	33.71	36.31	.
NEG	NEG	NEG	27.22	30.16	33.93	NEG	36.57
NEG	NEG	NEG	26.56	30.47	35.48	35.55	35.36
NEG	NEG	NEG	NEG	31.01	NEG	33.87	NEG
NEG	NEG	NEG	NEG	30.80	35.19	36.37	35.39
NEG	NEG	NEG	NEG	NEG	32.48	NEG	36.63
NEG	NEG	NEG	NEG	NEG	34.05	NEG	35.69
NEG	NEG	NEG	NEG	NEG	34.00	35.49	NEG
NEG	NEG	NEG	NEG	NEG	33.47	NEG	NEG
NEG	NEG	NEG	NEG	NEG	34.83	NEG	NEG
NEG	NEG	NEG	NEG	NEG	35.50	35.65	NEG
NEG	NEG	NEG	NEG	NEG	34.81	35.16	36.93
NEG	NEG	NEG	NEG	NEG	34.32	38.68	35.43
NEG	NEG	NEG	NEG	NEG	34.48	NEG	35.19
NEG	NEG	NEG	NEG	NEG	35.15	35.02	34.78
NEG	NEG	NEG	NEG	NEG	34.96	NEG	37.44
NEG	NEG	NEG	NEG	NEG	35.21	NEG	NEG
NEG	NEG	NEG	NEG	NEG	32.73	NEG	34.55
NEG	NEG	NEG	NEG	NEG	34.82	NEG	36.03
NEG	NEG	NEG	NEG	NEG	33.96	NEG	NEG

NEG	NEG	NEG	NEG	NEG	33.77	NEG	34.34
NEG	NEG	NEG	NEG	NEG	NEG	NEG	34.94
NEG	NEG	NEG	NEG	NEG	NEG	NEG	36.04
NEG	NEG	NEG	NEG	NEG	NEG	NEG	40.37
NEG	NEG	NEG	NEG	NEG	NEG	NEG	38.72

Supplemental Table S5. Data used for calculating the LOD95% using synthetic DNA (gBlock®) in the portable Q3NDPlus instrument. Concentrations are shown as copies/µL. NEG = negative

10^5	10^4	10^3	10^2	5x10^1	2.5x10^1	10^1	10^0
20.1	24.5	25.2	30.9	33.9	34.4	33.8	NEG
20.8	25.2	26.8	28.0	35.1	34.8	NEG	NEG
19.7	23.8	25.9	28.6	34.9	NEG	33.0	35.5
20.0	24.0	26.2	29.0	34.4	NEG	34.8	NEG
20.9	22.0	28.7	29.0	35.3	35.0	34.6	NEG
20.0	22.6	29.4	31.3	32.4	NEG	NEG	NEG
21.0	22.5	29.1	30.1	34.9	NEG	33.3	NEG
20.3	25.5	27.7	31.9	NEG	NEG	NEG	NEG
22.2	25.8	27.7	32.3	34.4	35.1		37.8
22.4	27.4	27.5	30.0	34.8	37.5		
	26.7	26.8	31.1	NEG	35.8		
	26.9	22.8	32.5	NEG	36.3		
	26.3	22.8		33.4	NEG		
	26.0	22.6		32.3	36.9		
	25.4	22.6		33.3	NEG		
		29.4			NEG		
		29.9			NEG		
		29.0			NEG		
		29.5			NEG		
		29.3			32.8		
